# Whole Genome Sequencing Analysis of *Salmonella enterica* Serovar Typhi: History and Current Approaches

**DOI:** 10.3390/microorganisms9102155

**Published:** 2021-10-15

**Authors:** Wan Ratmaazila Wan Makhtar, Izwan Bharudin, Nurul Hidayah Samsulrizal, Nik Yusnoraini Yusof

**Affiliations:** 1 Reconstructive Sciences Unit, School of Medical Sciences, Universiti Sains Malaysia, Kubang Kerian 16150, Malaysia; wrwm@usm.my; 2 Department of Biological Sciences and Biotechnology, Faculty of Science and Technology, Universiti Kebangsaan Malaysia UKM, Bangi 43600, Malaysia; ibb@ukm.edu.my; 3 Department of Plant Science, Kuliyyah of Science, International Islamic University Malaysia, Kuantan 25200, Malaysia; hidayahsamsulrizal@iium.edu.my; 4 Institute for Research in Molecular Medicine (INFORMM), Health Campus, Universiti Sains Malaysia, Kubang Kerian 16150, Malaysia

**Keywords:** whole-genome sequencing, *Salmonella enterica* serovar Typhi (*S*. Typhi), typhoid, virulence, multidrug-resistant, comparative genomics

## Abstract

In recent years, the advance in whole-genome sequencing technology has changed the study of infectious diseases. The emergence of genome sequencing has improved the understanding of infectious diseases, which has revamped many fields, such as molecular microbiology, epidemiology, infection control, and vaccine production. In this review we discuss the findings of *Salmonella enterica* serovar Typhi genomes, publicly accessible from the initial complete genome to the recent update of *Salmonella enterica* serovar Typhi genomes, which has greatly improved *Salmonella enterica* serovar Typhi and other pathogen genomic research. Significant information on genetic changes, evolution, antimicrobial resistance, virulence, pathogenesis, and investigation from the genome sequencing of *S*. Typhi is also addressed. This review will gather information on the variation of the *Salmonella enterica* serovar Typhi genomes and hopefully facilitate our understanding of their genome evolution, dynamics of adaptation, and pathogenesis for the development of the typhoid point-of-care diagnostics, medications, and vaccines.

## 1. Introduction

In humans, salmonellosis causes enteric fever, which could present as typhoid or paratyphoid and non-typhoidal gastroenteritis [1]. Typhoid is a considerable global public health issue that is an acute and a life-threatening disease infection caused by *Salmonella enterica* serovar Typhi (*S*. Typhi) and *Paratyphi* [2]. Salmonellosis is most common in developing countries, and Asia, Africa, South America, and South Asia are the regions most vulnerable to infection caused by multidrug-resistant *Salmonella* [3]. The disease is most common in the world’s least-developed tropical region, which also includes the least-developed areas in the world due to its fecal-oral transmission pathway. More than 80 percent per year of the world’s 12 million typhoid cases, especially among children and teenagers, occur in Asia and Africa. In Southeast and Central Asia, it has been reported that 200,000 fatalities and 22 million sicknesses per year are caused by enteric fever produced by *S*. Typhi and *S. Paratyphi* strains [4]. Additionally, typhoid fever may lead to long term physical and mental disorders if untreated over a long period [5]. These disease-causing pathogens are highly resistant to antibiotics, such as trimethoprim-sulfamethoxazole, ampicillin, and chloramphenicol [6]. Furthermore, a major economic burden is caused by a large number of typhoid fever cases in developing countries [7].

The *Salmonella enterica* species is composed of pathogenic bacteria that can infect many humans and animals, which causes various syndromes of disease [1]. *Salmonella* has been studied since the dawn of microbiology due to the diversity of antigens within the genus, resulting in the distribution of isolates to more than 2600 distinct serovars [4]. The classification of *Salmonella* was dominated by serological methods, which have demonstrated their effectiveness in both the clinical treatment of infections and epidemiologic monitoring. However, there are many subspecies and several serovars of *Salmonella* species that cause difficulties to taxonomists in defining the type accurately [1]. In recent years, modern genomics science and the capability to sequence the complete genome of bacteria have enhanced our knowledge of *S. enterica* species organization and evolution, which enables us to analyse specific members, such as *S*. Typhi [8,9,10]. A study of genome organization will assist in understanding the mechanism by which species evolve, and this will allow us to identify the types of organisms that will occur in the future [11].

Next-generation sequencing systems have made substantial advances in DNA sequencing technologies, providing higher accuracy and considerably lower costs [12,13]. The number of whole genomes stored in public repositories, such as the Online Genome Database (https://gold.jgi.doe.gov/, accessed on 15 July 2021), has grown exponentially due to next-generation technologies. Comparative analyses, such as pan-genomic analysis, have become possible with the great number of genomes available, particularly the prokaryotic genomes which drive gene discovery in the biomedical, biotechnological, and environmental fields [14,15]. Comparative genomics was used to examine intrinsic genomic features in other species. The virulence mechanisms in pathogenic organisms may be elucidated through the pan-genomic method by using multiple organisms of a single species or genera for the identification of similarities between genomes [16,17]. This approach can map the occurrence of and establish phylogenetic relationships for evolutionary events [18]. Furthermore, comparative genomics can be employed in microorganisms with different habits to compare their gene repertoires and genome sizes as intracellular pathogens often encounter reduced evolution and gene loss [19].

Progress in whole genome sequencing (WGS) technology has contributed to the high-throughput sequencing of bacterial genomes at reasonable prices, such that WGS has become an alternative to conventional outbreak typing and identification methods in public health [20]. Although WGS offers the possibility of resolving bacterial strains at the specific nucleotide resolution required to classify cases with a common infection source [21], categorizing isolates into higher taxonomic variants (e.g., those identified by serotyping) is a significant step. Public Health England (PHE) has successfully implemented WGS research for *Salmonella* Typhi, which substitutes the traditional serotyping method of *Salmonella* for regular public health surveillance and gives information on the genetic population distribution of *Salmonella* species in the world [22].

*S*. Typhi has an approximately over 5 million base pair-long genome and codes for over 4000 genes, from which more than 200 genes are actively dysfunctional. A global comparison study of *S*. Typhi isolates demonstrates that they are closely related and evolved from a single point of origin, about 50,000 years ago. Different strains of *S*. Typhi have plasmids that retain extrachromosomal DNA carrying virulence or antimicrobial resistance genes [11]. In this review, we discuss the historical research and the current and future impacts of WGS on *S*. Typhi. We highlight the initial attempt at performing WGS on *S*. Typhi using early sequencing technologies.

## 2. The Early Study of *S*. Typhi Whole Genome Sequencing Analysis: Comparison Study of *S*. Typhi and Other Bacteria

Different studies have been conducted to establish a safer remedy for *Salmonella* infections, such as species recognition, DNA and protein identification, and various biological techniques. As early as 1992, pulsed-field gel electrophoresis (PFGE), IS200 printing, ribotyping, and amplified fragment length polymorphism (AFLP) were used to recognise genetic variation within the *S*. Typhi community [23]. Four sequence forms were defined via the multilocus sequence typing (MLST) technique: ST1, ST2, ST3, and ST8. Both the ST1 and ST2 sequences were isolated from global sources or by only one polymorphism, while ST3 and ST8 were only isolated once each. The MLST technique and microarray-based comparative genomic hybridization (M-CGH) analysis also discovered the origin of *S*. Typhi genotypes for isolates obtained in China in 1959–2006. The *S*. Typhi genotypes studied belonged to four sequence groups from these reports: ST1, ST2, ST890, and ST2, the most prevalent genotypes of *S*. Typhi in China [24].

The WGS of *S*. Typhi began in 2002, when the first complete *S*. Typhi CT18 genome was released [23]. Parkhill et al. [10] successfully sequenced the *S*. Typhi CT18 which contains a 4,809,037-base pair genome that encodes genes of resistance to multiple drugs, showing the existence of hundreds of insertions and deletions compared to the genome of *Escherichia coli*, varying in size from single genes to large islands. A comparison of the *S. enterica* serovar Typhimurium (*S*. Typhimurium) strain LT2 and the *S.* Typhi strain CT18 revealed that both species shared approximately 89 percent of genes, with ≈480 genes found to be unique to *S.* Typhimurium, and ≈600 genes found to be unique to *S.* Typhi [25]. According to Sabbagh et al. [26], each species evolves through horizontal gene transfer or gene degradation mechanisms. In addition, genome sequencing analysis has resulted in discovering over 200 pseudogenes, many relating to genes believed to contribute to *S*. Typhi virulence. This genetic degradation can lead to *S.* Typhi’s human-restricted host range which can be found in phages, *SPI* genes, fimbriae and other virulence factors. In addition, the CT18 strain was also found to contain a 106,516-bp cryptic plasmid (pHCM2) and a 218,150-bp multiple-drug-resistance IncH1 plasmid (pHCM1) that show a common origin with a virulence plasmid of *Yersinia pestis* [10].

The second complete genome of *S.* Typhi, Ty2, was reported to have been isolated from Russia in the early 1970s [10]. The Ty2 strain is the basis for the production of vaccines, and is the parent of the Ty21a and CVD908 mutant strains whose descendants were used in live attenuated vaccine trials [9]. The size of the circular chromosomes of Ty2 and CT18 are also slightly different, of which Ty2 has 4,791,961 bp with an average G+C content of 52.05%, while CT18 has a total circular chromosome size with 4,809,037 bp with G+C content of 52.09% [9,10] (Table 1). In its population, *S.* Typhi also revealed a richness in genome composition. *S.* Typhi was observed to have a higher level of chromosome rearrangement in wild-type strains relative to *S.* Typhimurium. Due to the recombination between *rrn* operons, which are deletions, duplications, translocations, and inversions, the chromosome rearrangements could be formed. It was necessary to establish the order of fragments in the wild strains of the *S.* Typhi genome through a partial digestion by I-Ceul (an endonuclease, which cuts within the *rrn* operons) and separation by PFGE. The PCR analysis was used to validate the order and orientation of the I-Ceul fragments studied by PFGE [27].

Various details of *S*. Typhi could be extracted through comparative studies with *S.* Typhimurium, i.e., phylogenetic association of *S.* Typhi and *S.* Typhimurium was studied based on 16S rRNA sequence analysis. Through this analysis, pairwise evolutionary distances were calculated from the similarity values of 16S rDNA. A phylogenetic tree based on the 16S rDNA gene sequence from both species was constructed [28] using the distance matrix method of De Soete [29] and neighbour joining analysis [30]. The result shows that *S*. Typhi and *S*. Typhimurium strains have 99.7% similarity and were positioned in a phylogenetic cluster that contains *Enterobacter cloacae*, *E.* *coli*, and *Citrobacter freundii* (96.3 to 97.6% similarity). The analysis of 16S rDNA of *Salmonella* species and those of the *E. coli* strains are almost alike, thus the sequence makes it impossible to differentiate using phylogenetic relationships [28].

In 2008, the complete genome sequences for 19 *S*. Typhi isolates, including CT18 and Ty2, were generated using 454 (Roche, Indianapolis, IN, USA) and Solexa (Illumina, San Diego, CA, USA) technologies [31]. The comparative analysis found no evidence of purifying, antigenic variance, or recombination between the isolates. In comparison to strong adaptive selections of mutations that confer antimicrobial resistance in *S.* Typhi, there is little evidence for antigenic variation caused by immune selection. The results have also reported that evolution of *S.* Typhi appears to be characterized by the continuous loss of the gene function and a limited, effective population size. Furthermore, the comparison of entire genome provides a broad understanding of the range of genetic variants in *S*. Typhi, comprising single nucleotide polymorphisms, insertions, deletions, recombinations, plasmids and phage content [31]. Until 2010, it was recognized that species like *S. enterica* are continuing to evolve, and just a little snapshot is being observed as it changes over time. 

In short, the investigation of genome organization can help us to better comprehend how species develop, and it can also enable us forecast what type of organisms may eventuate in the future. Moreover, the application of next-generation sequencing (NGS) to the whole-genome sequencing of infectious illnesses, particularly *S*. Typhi genomes, has permitted a greater understanding of the pathogen’s evolutionary trends and its pathogenesis. This opens the door for the creation of efficient typhoid vaccines and treatments, which brings us closer to disease elimination.

## 3. The Middle Era of *S*. Typhi Whole Genome Sequencing Analysis: Detection of *S*. Typhi’s Variants

**Figure 1:**
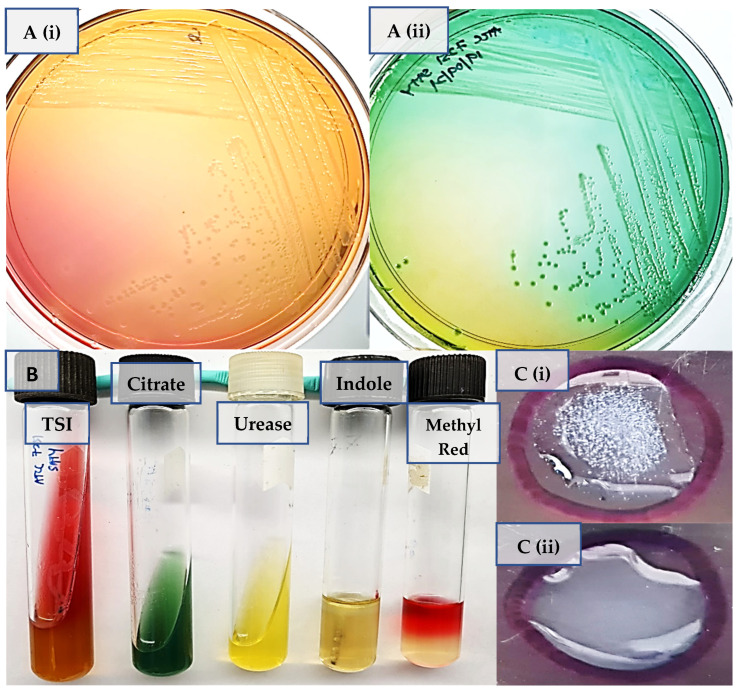
The initial step of diagnosing *Salmonella* species. (**A**) Two different culture agar media for identification of *S*. Typhi. (**A**) (i) The culture of *S.* Typhi colonies on the MacConkey agar is colourless due to the lack of lactose fermentation. (**A**) (ii) *S*. Typhi colonies which are blue-green, typically with black centers from hydrogen sulfide (H_2_S) gas, shown on Hektoen Enteric Agar. (**B**) Biochemical test for *S*. Typhi. The triple sugar iron (TSI) result showed an alkaline slant (red) and an acid butt (yellow) for the H_2_S production that resulted from the reduction in the sulfur component of the *S*. Typhi. No gas was produced. The data for Citrate, Urease, and Indole displayed negative, while Indole and Methyl Red displayed positive results for *S*. Typhi. (**C**) Serotyping is conducted from the determination of a fresh and pure culture of *Salmonella* on an agar medium. (**C**) (i) Agglutination was detected that indicated the development of serum antibodies when *S*. Typhi is tested to the O = 9.12 somatic, H = d flagellar, and “Vi” capsular antigens [37]. (**C**) (ii) No agglutination was observed, indicating the sample was not *S*. Typhi.

The invention of numerous sequencing technologies has rapidly increased the WGS data. In April 2015, PHE managed to implement WGS as the routine method for monitoring *Salmonella* infections in public health [22]. The initial step of diagnosing *Salmonella* infection from stool, body tissue, or fluid specimens is by using culture and biochemical tests (Figure 1A,B), followed by PCR [22]. The isolates were categorized into serovars according to the White–Kauffman–Le Minor (Figure 1C) scheme which had previously been used routinely at PHE [32,33]. The White–Kauffman–Le Minor scheme approach was developed for the detection of rabbit antisera reactions to lipopolysaccharide and flagellar antigens. This phenotypic variance expressed as an antigenic formula is used in the White–Kauffman–Le Minor scheme to distinguish *Salmonella* into over 2600 serovars. Over more than 70 years, serotyping (Figure 1C) has been used in the epidemiological analysis of *Salmonella* infection in humans and livestock. The serotyping method based on guidelines and regulations is recognized globally by national and international agencies [32,33]. However, traditional serotyping does not provide information on genetic relatedness and evolutionary perspectives between serovars [18].

The advent and implementation of WGS drastically revolutionized the techniques used not only to identify individual *Salmonella* strains, but also to detect precise types of serovars [18]. WGS is an economical typing method for outbreak detection and public health surveillance [20]. Furthermore, the advantage of WGS to address single nucleotide resolution bacterial strains demands the identification of related cases from a common source of infection and the categorization of the isolates into higher taxonomical levels (e.g., those defined by serotyping) in crucially important cases associated with a common source of infection [21]. Other than that, MLST can be predicted from WGS data, and as such has been proven to be a better alternative for *Salmonella* traditional serotyping for routine public health screening, and it has given an overview of *Salmonella* species’ genetic population in England and Wales in a twelve-month period [22].

The successful history of MLST identification via WGS data for the *S*. Typhi CR0044 genome was reported by Yap et al. [34]. The *S*. Typhi CR0044 was isolated from an asymptomatic typhoid carrier’s stool sample in Kelantan, in 2007 [35]. Using the MLST, this strain was subtyped as ST1, and was strongly related to the outbreak strain in 2005 analysed by PFGE. To sequence the genome of the *S*. Typhi strain CR0044, the Illumina Genome Analyzer was used, and it produced 1.0 gigabytes of data with depth coverage and read length of 90× and 73-bp, respectively. A genome de novo assembly using Velvet [36] resulted in the production of 201 contigs with a minimum of more than 200 bp contig length and a mean size of 23,367 bp.

The *S*. Typhi CR0044 genome is similar to the *S*. Typhi strains Ty2 and CT18, as both contain a type III secretion system and a flagellum sub-system [9,34]. The genes that contribute to the biofilm development and host persistence of cell walls, such as gene encoders for type 4 fimbrial assembly protein, the yjbRFGH locus, yhjD conserved clusters, and *wca* genes were also found in *S*. Typhi CR0044 [35]. However, this finding identifies that the genome differs from Ty2 and CT18 as it has the GGDEF family protein YeaJ, which is synonymous with the adhesion of the cell surface and the formation of biofilm [10,38]. The WGS of *S*. Typhi CR0044 also revealed the gene encoding the rhamnogalacturonide transporter RhiT for rhamnose utilization [39] and the azonular occludens toxin family protein, which was not previously reported in *Salmonella* spp.

The *S*. Typhi genome shows high genetic diversity, which exhibits genome variations and clonal expansion in Southeast Asia [40,41]. The ability of *S*. Typhi to survive in typhoid carriers is due to its naturally dynamic chromosome, which enriches its persistence and adaptation within the host. Carrier detection becomes particularly relevant since several of the traditional haplotypes in recent isolates have been found, indicating that these asymptomatic carriers are persistent [41]. The encoding of the gene *shdA*, which is the main factor expected to play a role in bacterial survival in the intestines, was detected through WGS of *S*. Typhi CR0063 isolated from a carrier in Kelantan during a long typhoid fever outbreak [42]. The gene product has the ability to attach to extracellular matrix proteins, fibronectin, and collagen by imitating host heparin, and presumably plays a key role in carriers by means of its long-term faecal shedding [42]. Interestingly, the *S*. Typhi CR0063 genome reveals strong similarities with the *S*. Typhi ST BL196 core genome regions found at the same time as a typhoid epidemic in Kelantan. Therefore, the genomic-level information was able to be used to unravel the evolution of the genome and the mechanism involved in the carrier-state transformation [42].

Comparative genomic studies have shown loss of gene function, which is known as pseudogenisation [43]. The latter is actively found in the human-restricted serovar *S*. Typhi compared to other serovars, such as *S*. Typhimurium [42]. In addition, even between host-restricted serovars, the extent of this pseudogenisation varies greatly [25,31]. Up to 4.5% of the gene pool of *S*. Typhi is pseudogenes, which makes them an important driver of genome re-assortment over time [31]. The eight strains of *S.* Typhi previously isolated from Southeast Asia and Oceania were associated with various medical symptoms, and were widely examined using a pan-genome technique. The pan-genomes of *S.* Typhi have been observed in their gene frequency in the functional and pseudogene complements, showing heterogeneity in its genome sizes. The result shows that the higher proportion of pseudogene is a more active process than a functional pan-genome. It also suggests that such a genome’s dynamic structure could contribute to its persistence and host adaptation [43,44].

Based on a report in 2012, during the 2005 typhoid outbreak in Kelantan, *S*. Typhi BL196/05 was isolated from blood samples drawn from an acute typhoid patient [42]. The Illumina 73-bp paired-end sequence read technology was used to generate 1.7 gigabytes of data, with 80 genomes covered. The assembly was performed using Velvet [36] to generate 191 contigs. The assembled sequence reads were submitted to rapid annotation-using subsystem technology (RAST) [45] for gene prediction. The analysis shows that the size of *S*. Typhi BL196/05 was approximately 4,744,056 bp with 53.21% of GC content (Table 1). Of the 4875 protein coding sequences, the genome contained 76 tRNA and 22 rRNA genes. The strain did not have any plasmids; however, its phage typing was identified as Vi phage type B1. Interestingly, the genome has two multiple antimicrobial resistance (*mar*) regulons, *marRAB* and *marC*, which is similar to *S*. Typhi strains CT18 and Ty2 and identical to the *E. coli* mar regulon members [9,46]. Other than that, genome analysis has revealed the melittin resistance protein *PqaB* and the polymyxin resistance protein *PmrD* was also found in the *S*. Typhi strain CT18 and Ty2 genomes. Furthermore, the rearrangements of the BL196/05 genome were correlated to the optimization of virulence, resilience, and host adaptation [44]. The insight of genome analysis revealed the genetic variety integration between *S*. Typhi isolates in Malaysia and Southeast Asia [47,48].

In Thailand, the typhoid fever outbreak from 1973 to 1976 made the government implement the national typhoid immunization program in 1977. In 2017, the consortium project had sequenced 44 *S*. Typhi isolates collected in Thailand between the outbreak (1973) and the end of the immunization program (1992) [47]. The immunization program greatly reduced the number of typhoid cases in Thailand. The results show that the isolates were highly diverse, especially after the immunization program, where the *S*. Typhi isolated were closely related to the strains isolated from bordering countries, such as Cambodia, Laos and Vietnam [47].

In general, the WGS data would provide an overview of *S*. Typhi variation, which is essential for the creation of new typing strategies, as well as for the improvement of established typing approaches, making it an excellent method for outbreak detection and epidemiological surveillance. The occurrence of the genome arrangement which promotes the diversity of *S.* Typhi has changed over time, was detected through WGS data analysis, and has contributed to improving tracking of the variants globally. 

## 4. The Current and Future Trend of *S*. Typhi Whole Genome Sequencing Analysis: Emergence of Antimicrobial Resistance *S*. Typhi Strains

The increasing cases reported globally, especially the emergence of multidrug-resistant (MDR) *Salmonella* have a huge impact on the global health population [48]. WGS has become a popular method of choice for *Salmonella* studies due to its high sensitivity and specificity for tracking and characterizing the transmission of *S.* Typhi [49], and for the prediction of antimicrobial resistance phenotypes, such as ampicillin, chloramphenicol, co-trimoxazole, tetracycline and ceftriaxone [50,51]. A consortium from various countries has sequenced 1832 *S*. Typhi isolated between 1905 and 2013 from different continents [52]. Further analysis had identified a dominant MDR lineage (H58), which began 30 years ago and scattered all over Africa and Asia. Moreover, H58 has also ousted the antibiotic-sensitive isolates, thus changing the *Salmonella* population globally. Interestingly, a study has shown that there are multiple transfers of H58 strains, notably from Asia, thus making it difficult for the detection of the Africa strain due to the divergence of the H58 lineages [52]. Additionally, the H58 can substitute the endemic *S*. Typhi isolates and broaden the disease into new areas in Malawi [53].

Fluoroquinolones are commonly known as an effective therapy for typhoid fever because they have good bactericidal and tissue-penetrating properties [51]. The emergence of fluoroquinolone-resistant strains was due to multiple mechanisms of fluoroquinolone resistance in *S*. Typhi, including decreasing outer membrane permeability, efflux pumps, genetic mutations, and plasmid-mediated acquisition. The chromosomal mutations in genes that encode DNA gyrase (ie, *gyrA* and *gyrB*) [48] and topoisomerase IV (ie, *parC* and *parE*) are the predominant mechanisms of fluoroquinolone resistance in *S*. Typhi [51,54]. The evolutionary process and occurrence time of fluoroquinolone resistance mutations in *S*. Typhi was explored by Matono et al. [55] using WGS (Table 2). They discovered that the same three chromosomal mutations in the quinolone resistance-determining region (QRDR) were present in all 33 highly resistant strains. Their research approach allowed them to distinguish fluoroquinolone susceptibility mutation patterns as highly resistant strains, which would probably have 3 mutations, 2 in *gyrA* (S83F and D87N) and 1 in *parC* (S80I). Within the evolutionary perspective, Matono et al. [55] conducted a pioneer study that comprehensively investigated the fluoroquinolone resistance mutations in *S*. Typhi. From susceptible strains without *gyrA* and *parC* mutation, *S*. Typhi evolved with *gyrA* S83F and developed more highly resistant mutations in *gyrA* and *parC*. On the contrary, the phylodynamic analysis revealed the fact that *S*. Typhi was highly resistant, and it evolved around 7 to 14 years after the introduction to ciprofloxacin treatment [55]. The genetic characteristics of several mutations in highly resistant strains could contribute to the survival and dissemination of bacteria and would benefit their environmental stress response.

A clinical strain isolated from a tertiary care hospital in Rawalpindi, Pakistan, which shows resistance to several antibiotics used in the treatment like cephalosporins (cefepime) and several fluoroquinolones, such as ciprofloxacin, levofloxacin, and moxifloxacin has also been sequenced [5,56]. Interestingly, further analysis identified two putative plasmid sequences (IncQ1 and IncY) and several antimicrobial resistance genes, such as *bla_CTX-M-15_, bla_TEM1_*, *gyrA* S83F, and *qnrS1*, which could potentially explain the observed resistance to the listed antibiotics [56]. On the other hand, the massive typhoid fever outbreaks between 2010 and 2012 in Zambia led to the WGST of 33 *S*. Typhi isolates. None of the isolates had the globally prevalent IncHI1 plasmid replicon type that is commonly present in haplotype H58 [57]. Of the 33 isolates, 32 possessed mutations associated in the QRDR of gyrase, the DNA topoisomerase IV genes *gyrA, gyrB, parC* and *pare*, and the plasmid-mediated quinolone resistance (PMQR) genes *qnrA, qnrB, qnrC, qnrD, qnrS, qepA* and *aac(6′)-1b* (Table 2). Twenty-seven isolates had an IncQ1 plasmid replicon sequence, and five isolates possessed an IncFIB plasmid replicon. Of these isolates, only four IncQ1-positive isolates contained a chromosomally translocated antimicrobial resistance region through a horizontal gene transfer event. The comparative genomic analysis of these four strains with *S.* Typhi CT18 revealed a truncated *IncQ1* region with the existence of *repA* and *repC* in all four strains 31, 34, 54 and 71 of the plasmid DNA region [57]. The findings indicated that the plasmid IncQ1 replicon and the antimicrobial resistance islands were translocated from the ancestral IncH11 plasmid to similar locations in the chromosomes of Zambian *S.* Typhi strains [57].

Typhoid fever has unexpectedly increased in Australia, Canada, Denmark, Ireland, the United Kingdom, the United States, and Taiwan [58]. In Italy, the first extensively drug-resistant (XDR) *S*. typhi infection and the first incident of a paediatric infection outside Pakistan from a carrier with a history of travelling to Pakistan was reported [58]. The WGS analysis found that this isolate belonged to the haplotype H58 and encoded the resistance region in the chromosome. Additionally, it also contained a plasmid that encoded additional resistance genes, such as the *qnrS* fluoroquinolone resistance gene and *bla_CTX-M-15_* extended-spectrum β-lactamase (Table 2). The two plasmids (IncY and IncQ1) were detected from the isolate using the PlasmidFinder tool. It was found that the IncY plasmid had no mutation and was identical to the plasmid found in the Pakistan outbreak [58].

Liaquat et al. [59] identified the potential virulence factors of the *S*. Typhi isolated from Pakistan in 2004–2013. Based on their analyses, they found two major variations in *Salmonella* pathogenicity islands (SPIs) region, mainly a 134 kb of SPI-7 and SPI-10 [59]. Three genes, *tviA, tviB*, and *pilS*, which were known to be associated with SPI-7, were missing from some of the isolates (11%), which are major differences from the *S*. Typhi genome. Moreover, Tanmoy et al. [51] detected a new local lineage comprising the *bla_CTX-M-15_* gene, conferring resistance to ceftriaxone, and *qnr* gene resistance to ceftriaxone (Table 2). The genotypes of both isolates were different from previous XDR isolates in Pakistan, which suggests a diverse geographical origin of the antimicrobial resistance. The high-resistance isolates caused by independent mutations that may increase the risk of global spread [19] were shown by the presence of a *S.* Typhi strain isolated from a patient who has a history of travelling to Pakistan, where the XDR *S*. Typhi outbreak incidents started in 2016 [60].

The contribution of WGS to the assessment of the whole DNA sequence of a bacterium is a great method for surveillance. WGS contains a conclusive evidence on genotype and provides the best approach available for individual organism characterization. Katiyar et al. [17] conducted WGS for 133 clinical isolates of typhoid patients. They implemented a comprehensive study of antimicrobial resistance genes and any potential associations with their phenotypes could contribute to the selection of more accurate treatments. They found that all fluoroquinolone resistance strains have mutations in the *gyrA, gyrB, parC*, and *parE* genes. The *catA1* gene confers chloramphenicol resistance, while trimethoprim-sulfamethoxazole for the *dfrA7, dfrA15, sul1,* and *sul2* and *bla_TEM-116_/bla_TEM-1B_* genes confer resistance to amoxicillin. However, for ceftriaxone and cefixime, no resistance determinants were identified. The acquired antimicrobial resistance genes that conferred resistance to aminoglycosides, including *aac(6′)-Iaa, AAC(6′)-Iy, aadA1, aph(3”)-Ib, aph(6)-Id, strA*, and *strB*, were observed in 133 isolates. Twelve isolates harboured *tet(A), tet(B)* and *tet(R)* genes that provided resistance to tetracycline. Additionally, the multidrug resistance genes such as *baeR, emrb, H-NS, marA, mdfA, mdtK, msbA, acrA, emrR, kpnE, kpnF, marR, sdiA, crp, soxR*, and *soxS* were also found in 133 strains (Table 2). This research increases the current understanding of the fact that WGS can aid in predicting resistance genotypes, and their associations with phenotypic traits allows antimicrobial resistance determinants to be detected quickly, thus prioritizing antibiotic usage directly from the sequence. Moreover, pan genome analysis showed that a few core genes were involved in metabolic functions, and the accessory genes were enriched; thus, they are believed to be associated in the pathogenesis and antimicrobial resistance of the *Salmonella* strains [17].

In observing the descendants of many bacteria, including *S.* Typhi, the most important technique of genotyping is MLST, which enables the differentiation of isolate characterization using the internal housekeeping gene sequence fragments. The *S*. Typhi lineages have been defined based on the recognized sequence types of the 7-loci MLST scheme. In earlier studies, *S.* Typhi ST1 and ST2 were the most widespread worldwide [60,61]. By contrast, the *S*. Typhi lineages were first reported only as a few uncommon strains that includes the ST8-typed isolate (422mar92 from Zaire, Africa, 1992) [23] and ST3 [62]. A Malaysian researcher had successfully determined the highly virulent strain (widespread) and the poor dissemination strain of *S*. Typhi genome using the MLST method. A total of 1826 publicly available *S*. Typhi genome sequences from different endemic regions in the world were used in the MLST study [62]. The very rare STs were observed from the large-scale genome sequence screening, including ST8, ST2233, and ST2359. The most interesting finding was that there was a co-existing presence of ST1 and ST2 regions in the endemic samples, while the ST8 was limited to the African samples only. Furthermore, comparative genomic analyses show that the specific mutations that occurred in the virulence genes *flhB, sipC*, and *tviD* had an effect on the pathogenicity of the *S*. Typhi isolates [62].

In summary, the sequences generated from several WGS for *S*. Typhi have contributed significantly to determining mutations in the *S*. Typhi genome, leading to the emergence of antimicrobial-resistant strains of a pathogenic bacterium. Indeed, the persistence of mutations and the horizontal gene transfer of the genetic material of antimicrobial-resistant strains would contribute to the innovation, evolution and genome sequence variation among *S.* Typhi facilitating the pathogen’s adaptation to environmental changes. 

## 5. Conclusions

WGS is important in specifying the correct diagnosis for typhoid fever because its symptoms may mimic other infectious diseases, such as dengue, hepatitis, leptospirosis, and malaria, especially in endemic regions of tropical countries [63]. The generated sequence data from comparative genomics have become important in developing genetic markers for this disease. Goay et al. [64] compared genomic sequences between *S.* Typhi and other enteric pathogens. A total of 111 samples, including ten clinical isolates of non-*Salmonella*, 62 *Salmonella* non-Typhi, and 39 *S*. Typhi were tested in the study. Based on the analysis, six of *S*. Typhi genes were selected for PCR assays for specificity test in vitro. Interestingly, five genes, STY0307, STY0322, STY0326, STY2020, and STY2021, exhibited high specificity and sensitivity using the PCR method [64]. This clearly shows the importance of WGS in the discovery of new genetic markers and developing a standard diagnostic method for typhoid fever, which could provide better management for reducing fatalities around the world. Rapid, simple, cost-effective and improved diagnostics are urgently needed for pathogen detection in polluted food, water and healthy human carriers to ensure the proper surveillance and monitoring of control initiatives. WGS is invaluable for finding biomarkers in typhoid carrier strains that require comprehensive evaluation using experimental methods. However, the experimental approach is challenging due to the lack of a human disease-reflecting animal model and high costs. Although researches have reportedly begun to investigate the typhoid carrier-state mechanism, it is still widely unexplored, and thus a great deal of work remains to be done to unravel our complicated understanding.

Nowadays, WGS technology has become more accessible to researchers due to its reasonable cost and rapidity. Conversely, the burden now lies in the analysis of the enormous amount of data obtained from WGS. For instance, Batool et al. [65] utilized a vast amount of data for a comparative and subtractive genomics study in identifying drug targets. They successfully identified 46 proteins which were crucial to the pathogen and missing in the host genome. A few screening processes via bioinformatic analysis had identified two enzymes (MurA and MurB) involved in the peptidoglycan synthesis pathway which were selected for 3D structure identification via homology modelling for molecular docking studies. The docking studies had identified a unique binding between the active site residues of the enzymes and the ligand atoms which could be chosen as the drug targets for typhoid fever [65]. This study required computational resources and bioinformatics expertise for whole data analysis. Thus, it is crucially important to develop a bioinformatics solution, focused on the development of simple analysis tools requiring only basic programming skills. Furthermore, a new pipeline on analysing the WGS data of *S*. Typhi could be a major focus of future work, since this will benefit other researchers with less programming knowledge. Moreover, artificial intelligence could be explored for designing more user-friendly bioinformatics tools for researchers, targeting those without programming skills backgrounds. 

In conclusion, with the emergence of sequencing technology, more WGS could be performed with new isolates, especially from newly reported cases. It is important to study the evolution and organization of the genome structure in different regions of the world utilizing large-scale comparative genomic analyses. Moreover, a focus on the noncoding regions of the genome is also important since they may be responsible for performing some of the most fundamental tasks in living cells. Furthermore, a new pipeline on analysing the WGS data of *S*. Typhi could be one of the major directions of future research, since to do so will benefit other researchers. Additionally, data generation must also be supported by the experimental validation of the genes predicted and could focus on the functional characterization of the specific proteins causing pathogenesis, such as the effector proteins and the antimicrobial resistance mechanism. The remaining challenge is to clarify the relationship between the increasing genome sequence data and the phenotypic properties of *S*. Typhi isolates in a publicly accessible format that will benefit us as a clinical reference worldwide.

## Figures and Tables

**Table 1:** The properties of *S.* Typhi genome isolated during 2001–2014.

Strain	Source of Isolates	Status	Genome Size (Mb)	Protein Coding Sequence	Accession Number	Reference
Ty2	USA	Complete	4.8	4323	AE014613	[9]
CT18	Vietnam	Complete	5.1	4766	AL513382	[9,10]
CR0044	Malaysia	Draft	4.8	4884	AKZO00000000	[35]
CR0063	Malaysia	Draft	4.6	4946	AKIC00000000	[42]
ST BL196/05	Malaysia	Draft	4.7	4875	AJGK00000000	[42]

**Table 2:** The emergence of antimicrobial resistance genes was detected in *S.* Typhi isolates.

Source of Isolates	Antimicrobial Gene	Antimicrobial Gene Mutations	Resistance Type	Reference
Bangladesh	*gyrA*	D53N, S83F, S83Y, D87N, N529S, D87G, D87Y, A119E, D87A	Ciprofloxacin	[51]
	*gyrB*	S464F, S464Y	Ciprofloxacin	
	*parC*	E84K, S80R, D69A, T620M, E84G, S80I	Ciprofloxacin	
	*parE*	A364V, T447A, L416F, S339L, A365S, L502F, E460K	Ciprofloxacin	
	*bla_CTX-M-15_*	NR *	Ceftriaxone	
	*bla_TEM-1B_*	NR	Ampicillin	
	*catA1*	NR	Chloramphenicol	
	*dfrA7*	NR	Co-trimoxazole	
	*strA, strB*	NR	Streptomycin	
	*sul1, sul2*	NR	Co-trimoxazole	
	*qnrS1*	NR	Ciprofloxacin	
	*tet(A), tet(B)*	NR	Tetracycline	
Malaysia	*gyrA*	S83F	Fluoroquinolones	[54]
Japan	*gyrA*	S83F, D87N, S83Y, E84G, D420N	Fluoroquinolone	[55]
	*parC*	S80I, Glu84Gly	Fluoroquinolone	
	*parE*	Asp420Asn	Fluoroquinolone	
Pakistan	*gyrA,* *bla_CTX-M-15_*, *bla_TEM-1_*, *qnrS1*	S83F NR	Cefepime and fluoroquinolones (ciprofloxacin, levofloxacin, and moxifloxacin)	[56]
Zambian	*gyrA*,	D87N, S83Y	Quinolone	[57]
	*gyrB*, *parC*, *parE*,	NR	Quinolone	
	*qnrA*, *qnrB*, *qnrC*, *qnrD*, *qnrS*, *qepA*, and *aac*(*6*′)-*lb*	NR	Quinolone	
	*sul1, sul2*	NR	Sulfamethoxazole	
	*dfrA14*, *dfrA7*	NR	Trimethoprim	
	*catA1*	NR	Chloramphenicol	
	*strA, strB,* *∆aadA1*	NR	Streptomycin	
	*bla_TEM-1_*	NR	Ampicillin	
	*qucE*	NR	Sulfonamide	
Italy	*qnrS*	NR	Fluoroquinolone	[58]
	*bla_CTX-M-15_*	NR	Ceftriaxone	
		
India	*gyrA*	D87N, S83Y, D87Y, S83F, D87G	Fluoroquinolone	[17]
	*gyrB*	S464F, A574V	Fluoroquinolone	
	*parC*	E84G, E84K, S80I	Fluoroquinolone	
	*parE*	A364V, D420N, L416F	Fluoroquinolone	
	*acrB*	R717Q	Azithromycin	
		
	*TEM-1, bla_TEM-1B_, bla_TEM116_*	NR	Amoxicillin	
	*dfrA7*, *dfrA15*, *sul1*, *sul2*	NR	Trimethoprim-sulfamethoxazole	
	*aac(6′)-Iaa, AAC(6′)-Iy, aadA1, aph(3”)-Ib, aph(6)-Id,*	NR	Aminoglycosides	
	*strA, strB*	NR	Streptomycin	
	*tet(A), tet(B), tet(R),*	NR	Tetracycline	
	*catA1*	NR	Chloramphenicol	
	*mdtK*	NR	Acrifavin, doxorubicin and norfoxacin	
	*catA1*	NR	Chloramphenicol	
	*dfrA7, sul1, sul2*	NR	Trimethoprim-sulfamethoxazole	
	*baeR, emrb, H-NS, marA, mdfA, mdtK, msbA, acrA, emrR, kpnE, kpnF, marR, sdiA, crp, soxR*, and *soxS*	NR	Multidrug resistance	

* NR: Not reported

## Data Availability

Not applicable.
